# Effect of Rebonding on the Bond Strength of Orthodontic Tubes: A Comparison of Light Cure Adhesive and Resin-Modified Glass Ionomer Cement In Vitro

**DOI:** 10.1155/2017/8415979

**Published:** 2017-03-13

**Authors:** Monika Aleksiejunaite, Antanas Sidlauskas, Arunas Vasiliauskas

**Affiliations:** Clinic of Orthodontics, Lithuanian University of Health Sciences, Lukšos, Daumanto St. 6, LT-50106 Kaunas, Lithuania

## Abstract

The purpose of this study was to determine the impact of different enamel preparation procedures and compare light cure composite (LCC) and resin-modified glass ionomer (RMGI) on the bond strength of orthodontic metal tubes rebonded to the enamel. Twenty human molars were divided into two groups (*n* = 10). Tubes were bonded using LCC (Transbond XT) in group 1 and RMGI (Fuji Ortho LC) in group 2. The tubes in each group were bonded following manufacturers' instructions (experiment I) and then debonded using testing machine. Then, the same brackets were sandblasted and rebonded twice. Before the first rebonding, the enamel was cleaned using carbide bur (experiment II) and before second rebonding, it was cleaned using carbide bur and soda blasted (experiment III). Mann–Whitney and Wilcoxon signed-rank tests showed no significant difference between RMGI and LCC bond strengths in case of normal bonding and rebonding, when enamel was cleaned using carbide bur before rebonding. Enamel soda blasting before rebonding significantly increased RMGI tensile bond strength value compared to LLC (*p* < 0.05). LCC and RMGI (especially RMGI) provide sufficient bond strengths for rebonding of molar tubes, when residual adhesive from previous bonding is removed and enamel soda blasted.

## 1. Introduction

The acid-etching bonding technique introduced by Buonocore in 1955 has revolutionized the bonding procedure [1, 2]. It was adopted for bonding orthodontic brackets clinically by Newman in 1968. The bonding of orthodontic brackets and tubes instead of banding improved treatment results in orthodontics. It resulted in decreased gingival irritation, improved esthetics, easier plaque removal, and elimination of pretreatment separation [3]. For bonding, orthodontists use different enamel pretreatment methods such as bur, air abrasion, acid etch treatment, and different adhesive materials [4]. Although most of the bonding procedures provide clinically acceptable bond strengths, orthodontic bond failure, especially failure of orthodontic tubes in the molar region, is still a serious problem for clinical orthodontics [5, 6].

There are two major components responsible for the final bonding strength: bonding system with adhesive materials and enamel preparation procedure.

The prevalent adhesive material in contemporary orthodontic practice is light cure composite (LCC). The bond strength of the composite resin with phosphoric acid etching is high (20–25 MPa) and sometimes may damage the enamel after debonding [7]. Composite adhesive systems can cause enamel loss up to 10 *μ*m [8]. Since metal brackets are opaque, light activation for 20 seconds may not be enough, while light activation for 40 seconds can increase the bond strength approximately by 9-10 MPa and create excessively firm bonding in some enamel areas [9]. The other problem with composite resin bonded orthodontic brackets and tubes is decalcification of the enamel and white spot lesions. The alternative for LCC is provided by glass ionomer cements (GICs). The GICs are fluoride-releasing materials with antibacterial properties and can reduce enamel decalcification but in general are characterized by lower bond strength compared to LCC [10, 11]. Addition of small amounts of light-activated resin was found to be effective for improving the properties of the GICs [12]. The resultant material is known as resin-modified glass ionomer (RMGI), which was introduced in 1988 [13]. Similar to GICs, RMGIs have fluoride release but are less susceptible to moisture and dehydration during setting and demonstrate better physical properties and bonding strength [14].

The second important component responsible for bond failure in orthodontics is the procedure of enamel preparation for the bonding. It is known that the dental pellicle and bacterial plaque reduce the bond strength; therefore, the enamel has to be prepared before etching [15]. The tooth surface should be pumiced using a rubber cup with fluoride-free paste. Some authors recommend using air polishing to remove soft dental plaque [16]. Then, enamel etching with phosphoric acid is recommended. Self-etching materials have been introduced to reduce the bonding steps and simplify the procedure; moreover, they have shown some other advantages, such as reduced loss of enamel and prevention of saliva contamination [17]. Therefore, normal bonding procedure in orthodontics has clear recommendations, but recommendations for rebonding are scarce.

There is no universally accepted minimum clinical bond strength for orthodontic attachments. However, the bond strength should withstand normal orthodontic and masticatory forces and thus should be between 8 and 9 MPa. Despite the fact that the acid-etching technique is a reliable bonding procedure in orthodontics, bracket and particularly molar tube failure are still serious problems for the clinical practice. The failure of orthodontic attachments is reported to be from 14 to 20% in cases using GIC and approximately 6–12% in cases using LCC [11]. Some clinicians prefer to use molar band after tube failure, instead, or rebonding the tube. However, this change is expensive and compromises the oral hygiene status. The rebonding of failed molars tubes could be a clinically and economically reasonable decision, but rebonding procedure should guarantee clinically acceptable bond strength. The data and practical recommendations regarding rebonding of orthodontic tubes are limited [18]. The purpose of this study was to determine the impact of different enamel preparation procedures and different bonding materials (LCC and RMGI) on the bond strength of orthodontic tubes rebonded to the enamel.

The null hypotheses to be tested were as follows:

1. There is no difference in the bond strength between the LCC and RMGI groups, whether the same molar tube is bonded for the first time or rebonded.
2. There is no difference in the bond strength of rebonded molar tube whether enamel from residual bonding material before rebonding was cleaned with a bur or with bur plus soda blasting.

## 2. Materials and Methods

Twenty mandibular molars with no caries, enamel cracks, or any other kind of damage and no pretreatment using chemicals (e.g., bleaching) were collected over a 2-month period. The extracted teeth were immersed in distilled water at a temperature of 37°C for 24 h. The teeth were then mounted into acrylic blocks (1 cm × 1 cm × 3 cm).

The archwires 0.21 × 0.25 TruForce SS (Ormco, Orange, California, USA) were inserted into the stainless steel molar tubes 0.22 Accent Mini (Ormco, Orange, California, USA), base surface area of 19.99 mm^2^, and bent into “U” shape. The wire was used to transmit the tensile force from the testing machine to the bonded tube.

**Figure 1:**
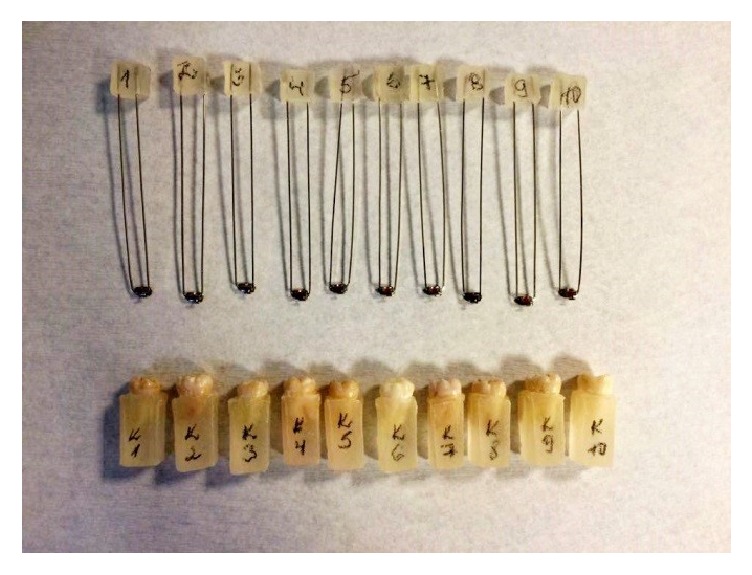
The teeth in acrylic blocks and archwire attached to the molar tubes.

The teeth were randomly divided into two groups. The teeth and the molar tubes were coded according to the randomly assigned group and numbered within the group (Figure 1).

The light cure composite Transbond XT (3M Unitek, Monrovia, California, USA) was used for bonding in group 1 and resin-modified glass ionomer cement Fuji Ortho LC (GC Corp., Tokyo, Japan) was used in group 2. A mounting jig was used to align the bracket base to be perpendicular to the bottom of the mold and parallel to the force during the strength test.

The bonding procedure was carried out strictly according to the manufacturers' recommendations (Table 1).

To test our hypotheses, the same molar tube was bonded thrice to the same tooth in every group.

*Experiment I (Initial Normal Bonding).* The molar tube was bonded to the clean tooth surface according to the manufacturer's recommendations and tensile bonding strength was measured by pulling it until debonding.

*Experiment II (First Rebonding).* The tube used in experiment I, checked for no deformation in the tube base, was sandblasted with Dentablast Duo (Dentaurum, Ispringen, Germany) using 50 *μ*m Al_2_O_3_, pressure 50 Ba, and 5 mm distance to remove the adhesive material from the surface with visual observation of the resin removal. The enamel of the tooth, after tube removal following normal bonding (experiment I), was cleaned up from adhesive material using a carbide bur (speed 15.000 rpm/min). The tube used in experiment I was rebonded and tensile bonding strength was measured by pulling it until debonding. 

*Experiment III (Second Rebonding).* The tube used in experiments I and II, checked for no deformation in the tube base, was sandblasted with Dentablast Duo (Dentaurum, Ispringen, Germany) using 50 *μ*m Al_2_O_3_, pressure 50 bar, and 5 mm distance to remove the adhesive material from the surface with visual observation of the resin removal. The enamel of the tooth, after experiment II, was cleaned up from adhesive material using carbide bur (speed 15,000 rpm/min) and soda blasted for 3 s with PROPHYflex 3 powder blast handpiece (Kavo, Biberach, Germany) using Prophy Powder (WP Dental, Barmstedt, Germany) at 2 mm distance. The used tube was rebonded for the second time and the tensile bonding strength was measured by pulling it until debonding.

**Figure 2:**
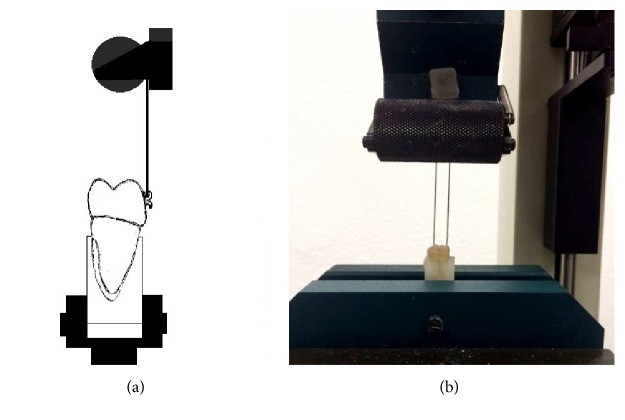
The scheme of the experiment (a) and the experiment in the laboratory (b).

The bond strength was tested using Tinius Olsen Testing Machine with a crosshead speed of 100 mm/min and worked until the bracket debonding (Figure 2).

Data were analyzed using IBM SPSS Statistics 23.0 software, while comparisons and contrasts were made using nonparametric Mann–Whitney and Wilcoxon signed-rank tests. The power of the research was 0.79, while type I error rate was 0.05.

## 3. Results

The descriptive statistics comparing the molar bond strength of the two groups in the three experiments are shown in Table 2.

**Figure 3:**
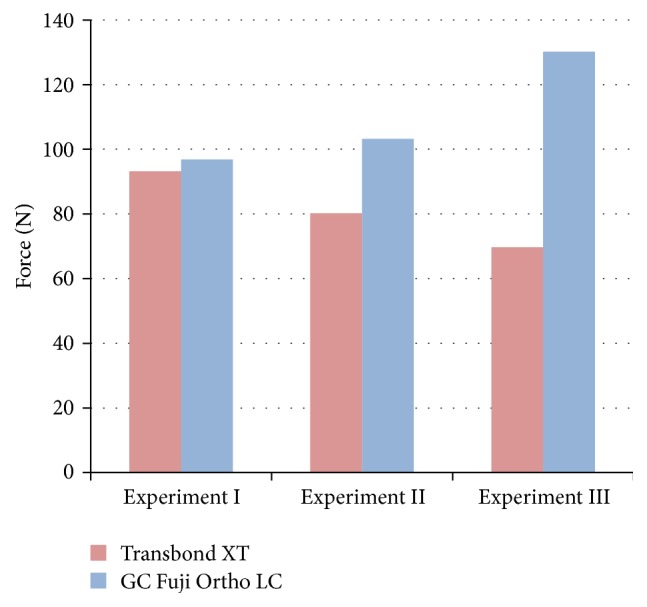
The bond strengths of the light cure composite and glass ionomer cement.

The RMGI demonstrated a bond strength similar to that of LCC in general and an even higher strength (*p* < 0.05) in experiment III (Figure 3).

Wilcoxon signed-rank test was used to assess the impact of the molar tube rebonding procedure on its tensile bond strength.

**Figure 4:**
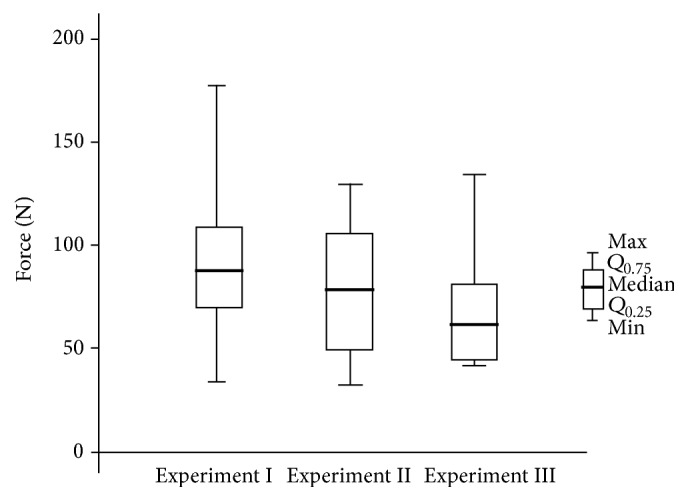
The effect of rebonding on the molar tube tensile bond strength. Bonding material: LCC,* Transbond XT*.

*In group 1 (bonding material: light cure composite, Transbond XT),* rebonding with LCC did not affect the bond strength significantly. The bond strength has a tendency to reduce after rebonding, but the difference was not statistically significant between the initial normal bonding and subsequent rebonding (*p* > 0.05) (Figure 4).

**Figure 5:**
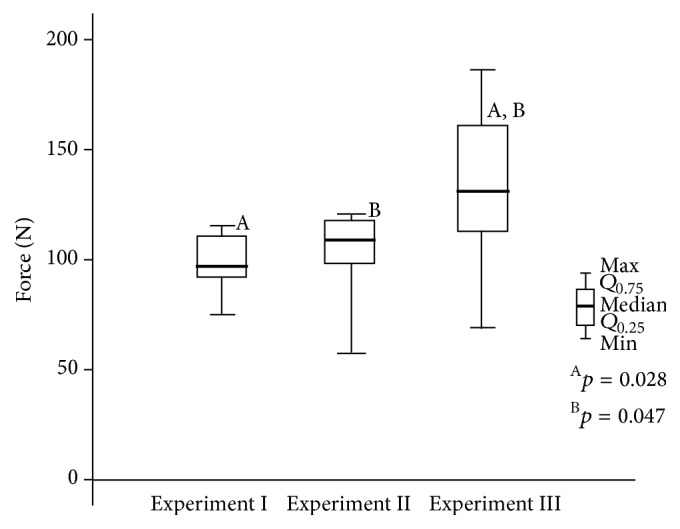
The effect of rebonding on the molar tube tensile bond strength. Bonding material: RMGI,* Fuji Ortho LC.*

*In group 2 (bonding material: resin-modified glass ionomer, Fuji Ortho LC),* the bond strength after rebonding with RMGI was dependent on the enamel cleaning method from previous bonding residual material. Enamel cleaning with a carbide bur had no impact on the subsequent bonding (experiment II), but removal of residual adhesive with bur and soda blasting (experiment III) considerably increased the bond strength (Figure 5). The improvement was statistically significant not only between initial bonding and experiment III (*p* = 0.028), but also between the first and second rebonding (*p* = 0.047).

## 4. Discussion

The null hypotheses were accepted with some exceptions. The results of this study did not detect statistically significant differences in the bond strengths between the LLC and RMGI, as well as between the initial bonding and rebonding of molar tubes. The exception in the acceptance of the hypotheses was that, after the second rebonding of the molar tube following soda blasting, RMGI demonstrated higher bond strength compared to LCC and to previous bonding with RMGI itself.

The literature data regarding comparing the advantages and feasibility of RMGI and LCC for orthodontic bonding are controversial. Previous studies have reported poor properties of glass ionomers as orthodontic cements [11, 14, 17]. GICs have some drawbacks for orthodontic bonding, namely, weak bond strength [19], high rate of bracket detachment [20], and poor early mechanical properties [21]. The modification of glass ionomer cements by reinforcing with resin components (RMGIs) improved their bond strength [18]. Reynolds and von Fraunhofer [22] suggested that minimum bond strength of 5.9–7.8 MPa is required for bracket bonding to enamel surfaces, while Lopez [23] showed that the shear bond strength of 7 MPa provides clinically successful bonding. The bond strength of RMGIs to enamel ranges from 5.4 to 18.9 MPa, as reported in the orthodontic literature; thus, it could be suitable for orthodontic bracket and tube bonding [24–26]. Yassaei et al. used exactly the same materials as our study, Transbond XT and Fuji Ortho L. They found that RMGI has significantly lower bond strength compared to composite resin [27]. On the contrary, the results of our study indicate that RMGIs provide bond strengths within the clinically acceptable ranges and even higher than LCC, when enamel is soda blasted before bonding. The possible explanation of this result could be that soda blasting reduces bacterial plaque and increases enamel porosity. The RMGIs can then penetrate deeper into the enamel tubes and more calcium in the tooth structure expresses affinity to carboxylate groups with the reacted RMGI. Pakshir et al. divided 50 upper premolars into two equal groups on the following basis: in group I, the enamel surface was etched with 37% phosphoric acid and in group II, the teeth were sandblasted prior to acid etching. Transbond XT adhesive material was used for bonding in both cases. The bond strength in group I (158.01 N) was significantly lower (193.44 N) than that in group II [28]. Halpern and Rouleau used a special soda powder for air abrasion, which is popular in oral hygiene procedures. They divided 212 human lower premolars into four equal groups: group 1 underwent no air abrasion (control group), group 2 received treatment with 25 *μ*m aluminum oxide particles, group 3 received treatment with 50 *μ*m particles, and group 4 received treatment with 100 *μ*m particles. This study revealed that air abrasion increases bond strength [29]. This is in accordance with our study; soda blasting increases bond strength of orthodontic bonding.

Orthodontic debonding has another clinically important side. In their study, Jassem et al. revealed that enamel fracture could occur with bond strengths as low as 13.5 MPa [30]. Knösel et al. compared bond strengths of GIC and LLC (Mono-Lok2, Ormco) using ninety-six third molars and upper premolar metal brackets. They, like many other studies, found that glass ionomer cement bonded brackets were easier to remove than those bonded with LCC [2, 8, 20]. The study of Yassaei et al. shows that brackets bonded by means of Fuji Ortho LC differed from those bonded using Transbond XT adhesive in the sites of bond failure. More adhesive remained on teeth in brackets bonded with Transbond XT than in brackets bonded with Fuji Ortho LC. Bond failure for brackets bonded with Fuji Ortho LC occurred mostly at the enamel-adhesive interface, while brackets bonded with Transbond XT typically failed at the bracket-adhesive interface [27]. Bond failure at the enamel-adhesive interface leaves less adhesive remnants on the enamel surface and therefore decreases the risk of enamel damage during adhesive removal. Therefore, the results of our study demonstrate that rebonding with RMGI after soda blasting creates sufficient bond strength and possibly prevents enamel damage.

When comparing this study with other studies, the different experimental parameters possibly influencing the results should be considered. Orthodontic tubes bonded to the molars were used in this study, while majority of similar studies used premolars and brackets. Therefore, tooth anatomy as well as bracket system and tube design can influence the accuracy of placement and retention. In addition, it is important to note that we used higher crosshead speed of the testing machine, compared to similar studies. The main objective for using higher speed was to imitate real dynamic forces of the masticatory process.

From a clinical standpoint, the use of RMGI for rebonding of molar tubes with soda blasting can be desirable, because it improves the adhesive strength and minimizes the risk of enamel damage in case of repeated debonding. However, this was a laboratory study and care should be taken in interpreting the results. Clinical studies are required to confirm the advantages of this recommendation in everyday practice.

## 5. Conclusions

Transbond XT and Fuji Ortho LC provide sufficient bond strength for rebonding of metal molar tubes. The enamel soda blasting significantly increased the rebonding strength of Fuji Ortho LC.

## Figures and Tables

**Table 1:** The bonding instructions for light cure composite and glass ionomer materials.

Bonding material	Application steps
Transbond XT (group 1)	(1) Cleaning of enamel surface for 30 s with nonfluoridated paste and rubber cups (Kerr Superpolish) (2) Enamel conditioning with 37% phosphoric acid for 30 s. Washed with water (30 s) and dried to a chalky white appearance (3) Primer application for 15 s (4) Adhesive application to tube base (5) Immediate tube positioning on enamel. Excess adhesives were removed with a sharp scaler (6) Light curing 10 s at each of the mesial, distal, occlusal, and gingival faces

Fuji Ortho LC (group 2)	(1) Cleaning of enamel surface for 30 s with nonfluoridated paste and rubber cups (Kerr Superpolish) (2) Enamel conditioning with GC Fuji Ortho conditioner for 10 s, washing with water, drying carefully with compressed air (1-2 s) (3) Mixing of fluid and powder according to the manufacturer's instructions (4) Immediate positioning of the coated tube base on the enamel, removal of excess adhesives with a sharp scaler (5) Light curing for 10 s at each of the mesial, distal, occlusal, and gingival faces

**Table 2:** The bond strength: mean values (*N*) and descriptive statistics.

Experiment	Group number	*n*	Mean (SD)	Min	Max
Experiment I	1	10	93.21 (41.32)	33.7	177.4
	2	10	96.88 (14.47)	74.7	115.5
Experiment II	1	10	80.25 (33.14)	32.0	129.6
	2	10	103.28 (18.9)	57.0	123.6
Experiment III	1	10	69.69 (32.05)	36.7	133.9
	2	10	130.23 (38.91)	68.6	185.6
